# Delivery Mode Affects Intestinal Microbial Composition and the Development of Intestinal Epithelial Cells

**DOI:** 10.3389/fmicb.2021.626144

**Published:** 2021-08-13

**Authors:** Linzheng Lyu, Xiaohong Zhou, Meng Zhang, Li Liu, Haiyue Niu, Jiliang Zhang, Shiwei Chen, Pimin Gong, Shilong Jiang, Jiancun Pan, Yuanyuan Li, Xue Han, Dayou Cheng, Lanwei Zhang

**Affiliations:** ^1^School of Chemistry and Chemical Engineering, Harbin Institute of Technology, Harbin, China; ^2^Qingdao Central Hospital, Qingdao, China; ^3^The Third Affiliated Hospital of Heilongjiang University of Chinese Medicine, Harbin, China; ^4^College of Food Science and Engineering, Ocean University of China, Qingdao, China; ^5^Heilongjiang Feihe Dairy Co., Ltd., Beijing, China

**Keywords:** delivery modes, intestinal microbiota, bacterial screening, infant intestinal epithelial cells, cell proliferation

## Abstract

**Background:**

The infant’s intestine contains diverse microbiota, which play an important role in an infant’s health.

**Objective:**

This study aimed to analyze the different intestinal microbiota and their function in two delivery modes [vaginal delivery and cesarean section (C-section)] and to investigate the proprieties of bacteria associated with vaginal delivery on the development of intestinal epithelial cells in rat pups.

**Materials and Methods:**

We evaluated the intestinal microbial diversity of the stool samples of 51 infants of subjects who underwent vaginal delivery and C-section by sequencing the V4 regions of the 16S rRNA gene and predicted the function of the microbiotas. The infant stool microbiota in the vaginal delivery group was associated with the digestive system and cell growth and death, whereas that of the C-section group was associated with membrane transport. Then, we isolated the strains based on function prediction.

**Results:**

A total of 95 strains were isolated in the vaginal delivery group. *Bifidobacterium bifidum* FL-228.1 (FL-228.1) was screened and selected owing to its good surface hydrophobicity, bacterial survivability in the simulated gastrointestinal condition and adhesion ability to the IEC-6 cell line as well as owing to the development of intestinal epithelial cells. Furthermore, *in vivo* experiments revealed that FL-228.1 exhibited favorable effects on the development of intestinal epithelial cells in rat pups.

**Conclusion:**

The results of this study indicate an apparent difference in the bacterial composition of the stool samples collected from infants of the two delivery modes. By analyzing and screening the bacteria in infant stool samples, we found that one strain, i.e., *B bifidum* FL-228.1, exhibited favorable effects on the development of intestinal epithelial cells.

## Introduction

The human intestinal microbiota comprises trillions of microbes and contains millions of functional genes that are mutually beneficial to the host (Friedrich, 2013). After birth, an infant’s intestine begins to be colonized by various microorganisms, including *Enterobacteria*, *Enterococci*, *Staphylococci*, *Bifidobacteria*, and *Lactobacilli* (Nagpal et al., 2017). These microbiota colonized on the intestine play an important role in infant growth and affect the child’s overall health status (Maldonadolobón et al., 2015).

Research has shown that the diversity and composition of the intestinal microbiota in early infants are greatly influenced by external factors (Madan et al., 2016b). Furthermore, many studies have reported that delivery mode is an important factor affecting the composition of the intestinal microbiota throughout the neonatal period and into infancy (Shao et al., 2019). During vaginal delivery, the microorganisms in the vagina are transmitted to the infants vertically. Therefore, Actinobacteria and Bacteroidetes are abundant at the phylum level, whereas *Bifidobacterium* is the dominant species at the genus level (Lundgren et al., 2018). Compared with vaginal delivery infants, the intestinal microbiota of cesarean section (C-section) infants predominantly originate from the mother’s skin as well as from microorganisms in the environment. Therefore, the relative abundance of Firmicutes and a few microbes from the skin, such as *Staphylococcus* and *Clostridium*, are higher than that of other bacteria (Jakobsson et al., 2014).

Studies in infants have indicated that C-section infants tend to have lower number of anaerobes (e.g., Bacteroidetes) and a less diverse microbiota than vaginal delivery infants (Chong et al., 2018). Further, the composition of microbiota in vaginal delivery infants is relatively more stable than that of C-section infants until 2 months of life (Reyman et al., 2019). Analysis of the intestinal microbiota of infants has revealed that there are differences in the composition of the intestinal microbiota of C-section and vaginal delivery infants (Shao et al., 2019). Interestingly, although there are apparent differences in stool microbiological composition of C-section and vaginal delivery infants after birth, a study has shown that C-section subjects gradually progress toward harboring microbiota reassembling natural birth infants by week 8 of life, which is maintained till week 24 (Hill et al., 2017).

Previous studies have compared the bacterial composition of stool samples of C-section and vaginal delivery infants (Madan et al., 2016b). Epidemiological evidence has revealed that C-section affects the initialization of intestinal microbiota colonization in infants, which in turn affects the formation and maturation of the intestinal immune system (Planer et al., 2016). A study by Salam (Salam et al., 2006) reported that C-section damages intestinal immunity by increasing the risk of gastrointestinal infections and allergies. However, to the best of our knowledge, no study has screened and isolated bacteria based on these two delivery modes for functional exploration. As a result, this study aimed to analyze the different intestinal microbiota and their function in the two delivery modes as well as to investigate the proprieties of bacteria associated with vaginal delivery on the development of intestinal epithelial cells in rat pups.

## Materials and Methods

### Participants

Fifty-one healthy families were recruited from the Third Affiliated Hospital of Heilongjiang University of Chinese Medicine (China) and Harbin Children’s Hospital (Harbin, China). The Institutional Review Board of Harbin Children’s Hospital approved this study; written informed consent was obtained from all the mothers. Prior to sampling, we surveyed the mothers via a questionnaire to collect clinical data. Infant stool samples were collected at the hospital or during a home visit in a period of 30 days. They were promptly transported to the laboratory and stored at –80°C until further analysis (Bäckhed et al., 2015).

### Isolation of Different Strains

One gram of the stool sample was diluted with 9 mL of phosphate-buffered saline (PBS buffer). The semi-selective medium method was used to screen the strains, as shown in Table 1. The plates were anaerobically incubated at 37°C for 72 h and then left in the air for 4 h. Single, blue colonies were isolated and subcultured for further purification and experiments. Each selected strain was subjected to microscopic examination and gram staining. The gram-positive strains were selected for further research (Riaz Rajoka et al., 2017).

**TABLE 1:** Semi-selective medium.

Putatively common strains	Additive>	Semi-selective medium (Martín et al., 2009; Lau et al., 2016)
*Lactobacillus*	Nalidixic acid and X-gal	MRS-Cys/LBS-Cys
*Bifidobacterium*	Nalidixic acid and X-gal	MRS-Cys/LBS-Cys

### Surface Hydrophobicity

The surface hydrophobicity of the selected strains was determined using a previously described method with some modifications (Xu et al., 2018). Briefly, the bacteria were incubated at 37°C for 72 h, centrifuged for 3 min at 6000 r/min and then resuspended in 3 mL of sterile PBS buffer. The absorbance of the suspension was measured at 600 nm (A_0_). Then, the suspension was mixed with 1 mL of ethyl acetate and placed at room temperature for 10 min. The two-phase system was mixed via swirl oscillation for 2 min. Then, the two-phase system was reconstructed by standing for 20 min. The aqueous phase was carefully removed, and the absorbance was measured at 600 nm (A_1_). The reference strain *Lactobacillus rhamnosus* LGG (ATCC 53103) was used as a control. Surface hydrophobicity was calculated according to the following formula (Pan et al., 2006):

H% = (1−A_1_/A_0_) × 100

### Bacterial Survivability in a Simulated Gastrointestinal Condition

The isolates were incubated in a simulated gastric fluid of PBS buffer supplemented with pepsin (0.5%) and adjusted to pH 2.5 with 0.1 M hydrochloric acid. After incubation at 37°C for 1.5 h, the viable isolates were evaluated via plate count on MRS agar. The simulated intestinal juice tolerance of the isolates that survived for 2 h in the simulated intestinal juice was determined using PBS buffer containing 0.3% (w/v) bile salt and trypsin (1 mg/mL); the pH was adjusted to 8.0. Following a 2-h incubation at 37°C, the simulated intestinal juice was assessed by measuring the survived isolates using MRS agar medium (Xu et al., 2018). The reference strain *Lactobacillus rhamnosus* LGG (ATCC 53103) was used as a control.

### Bacterial Adhesion Ability to the IEC-6 Cell Line

IEC-6 (National Collection of Authenticated Cell Cultures) were incubated in RPMI-1640 medium supplemented with 10% heat-inactivated fetal bovine serum. For adhesion assays, IEC-6 cells were seeded into 24-well tissue culture plates at a concentration of 2.5 × 10^5^ cells/well and incubated at 37°C for 24 h in 5% CO_2_ and 95% air. Then, the bacteria were added into the corresponding wells. Following 4-h incubation, IEC-6 cells were washed with PBS buffer to remove the bacterial suspensions, followed by lysis of non-adherent bacteria with 0.1% Triton X-100 solution. The adherent bacteria were serially diluted and spread on MRS agar plates. The plates were incubated in an anaerobic environment at 37°C for 72 h to calculate the number of adherent bacteria. The reference strain *Lactobacillus rhamnosus* LGG (ATCC 53103) was used as a control. The adhesion percentage of the bacteria was calculated according to the following formula (Oh and Jung, 2015):

Adhesion ability rate (%) = [adhered cell number (log CFU/mL)/initial cell number (log CFU/mL)] × 100%

### Development of Intestinal Epithelial Cells *in vitro*

A concentration of 2.5 × 10^5^ cells/mL was inoculated into a 24 well plate; the culture medium was changed after overnight culturing at 37°C. Then, bacteria were added into the corresponding wells at the ratio of 1:100. The bacteria and IEC-6 cells were cultured together for 4 h, followed by washing IEC-6 cells with PBS buffer. The blood cell counting plate was used to count the number of IEC-6 cells in each well. The reference strain *Lactobacillus rhamnosus* LGG (ATCC 53103) was used as a control. The experiment was repeated three times.

### Identification of the Different Strains

The 16S rRNA gene sequencing method was used to identify the isolated strains. Briefly, genomic DNA was extracted using the E.Z.N.A. bacterial DNA kit (Omega Bio-Tek, Norcross, GA). The 16S rRNA gene was amplified using the universal primers 27F (5′-AGAGTTTGATCCTGGCTCAG-3′) and 1492R (5′-TACGGTTACCTTGTTACGACTT-3′) (Meng et al., 2018). The amplicons were sequenced by Sangon Biotech Co., Ltd. (Shanghai, China). Sequence similarity analysis was performed by comparing the sequences with those in GenBank via the BLAST search program of the National Centre for Biotechnology Information.^1^

### Establishment of the Intrauterine Growth Retardation (IUGR) Animal Model

Pregnant female Sprague Dawley rats were provided by Charles River Laboratory Animal Technology Co., Ltd. (Beijing, China). Pregnant rats were fed separately. The IUGR animal model was established via maternal nutritional restriction. Pregnant rats were randomly divided into two groups: Normal diet group and food restriction group. The rats in the normal diet group were allowed *ad libitum* access to food and water. From the 3rd day of pregnancy to the day of delivery, the rats in food restriction group were provided with 50% of food. Both groups received adequate food and water on the day of delivery. Compared with normal neonatal rats, IUGR neonatal rats were successfully modeled when the weight of neonatal rats was less than two standard deviations in the food restriction group. Following successful modeling, the models were grouped into the following groups, as shown in Table 2: Blank control group, positive control group and experimental group. Neonatal rats were naturally fed for 3 days. Then, the intervention experiment was conducted. The bacteria were supplemented to pups via oral gavage using a special syringe (0.6 mm diameter). Rat pups were anaesthetized with isoflurane and sacrificed via cervical dislocation on the 10th day (6 pups/group) (Ding et al., 2016).

**TABLE 2:** Intervention treatment.

Groups	Intervention treatment
Blank control group	Normal lactation and intragastric administration of PBS
Positive control group	Normal lactation and intragastric administration of LGG PBS suspension (10^9^ CFU/mL)
Experimental group	Normal lactation and intragastric administration of the FL-228.1 strain in a PBS suspension (10^9^ CFU/mL)

### Intestinal Morphological Characteristics of Rat Pups

The intestinal morphology of rat pups was assessed via the villus length to determine whether the intestinal structure was affected by bacterial supplementation using the hematoxylin-eosin -staining. Following dehydration, the fixed intestinal villus tissues were embedded in paraffin, sliced (5 μm), patched in warm water at 42°C and copied at 42°C for 8 h. The hematoxylin–eosin-stained paraffin sections were observed under a Zeiss Axio Imager microscope (Désir-Vigné et al., 2018).

### Effect of Strains on Intestinal Cell Development

The small and large intestinal tissues of the nuclear development marker Ki-67 were collected via immunostaining on the 10th day to detect the contribution of bacteria toward the changes in intestinal structures. The immunohistochemical method was used to detect the expression of the cell development activity indicator in the intestinal mucosa. Parallel tissue slices of the cells were taken. Rabbit anti-Ki-67 monoclonal antibody was incubated in a wet box at room temperature. HRP-labeled anti-rabbit IgG was incubated at room temperature for 1 h. The positive result of DAB staining was brown at the antigen location (Bhinder et al., 2017).

### Illumina Sequencing and Statistical Analyses

Total genomic DNA was extracted from infant stool samples using the E.Z.N.A. bacterial DNA kit (Omega Bio-Tek, Norcross, GA) following the manufacturer’s instructions. The V4 region of the 16S rRNA gene was sequenced on the HiSeq 2500 sequencer, which was performed by BGI Tech Solutions Co., Ltd. (Shenzhen, China). The microbiome data were then analyzed using the Mothur software (V1.31.2). All samples were analyzed at the operational taxonomic unit (OTU) level. An OTU was defined as a group of bacteria with more than 97% similarity. Differences in OTUs were analyzed in every sample via α-diversity (chao1 and Shannon indices) using the R package. The bacterial diversity of infant stool samples was analyzed using the R package (ade4). Community composition analysis between the groups was performed using distance matrices (Adonis, R vegan package) (Pragman et al., 2018). All linear discriminant analysis effect size (LEfSe) taxonomic information was detected using the Metaphlan 2 program, with the cladogram derived from LEfSe analysis (Liu et al., 2016). The Illumina sequences were submitted to the NCBI Sequence Read Archive database with an accession number of PRJNA683130. The 16S rRNA sequence of FL-228.1 was submitted to the GeneBank database with an accession number of MT071594.

All experiment results were analyzed with SPSS software (version 22). *P*-values less than 0.05 were considered statistically significant.

## Results

### Questionnaire and Subject Information

Fifty-one healthy infants were recruited; their basic information is summarized in Table 3. Among the infants recruited, there were 32 boys and 19 girls. In total, 25 (49%) infants were born via vaginal delivery and 26 (51%) were born via C-section.

**TABLE 3:** Basic information of participants.

Characteristics	Delivery modes			
	C-section		Vaginal	
	Median or No.	Interquartile ranges or percentage	Median or No.	Interquartile ranges or percentage
Mother’s age (y)	31	22–38	29	23–35
Father’s age (y)	33	25–43	29	24–37
Mother’s pregnancy weight (kg)	74.2	57–96	69.75	60.6–92.5
Gestation age (d)	277	266–284	279	266–288
Birth weight (g)	3725	3,000–4,800	3400	2,700–3,900
Birth length (cm)	52	49–55.6	50	49–51
Infant sex				
Male	18	69.23%	14	56.00%
Female	8	30.77%	11	44.00%
Feeding patterns				
Exclusive breastfeeding	16	61.54%	12	48.00%
Mixed feeding	10	38.46%	13	52.00%

### Biodiversity of the Intestinal Microbiota of Infant Born via Different Delivery Modes

**FIGURE 1:**
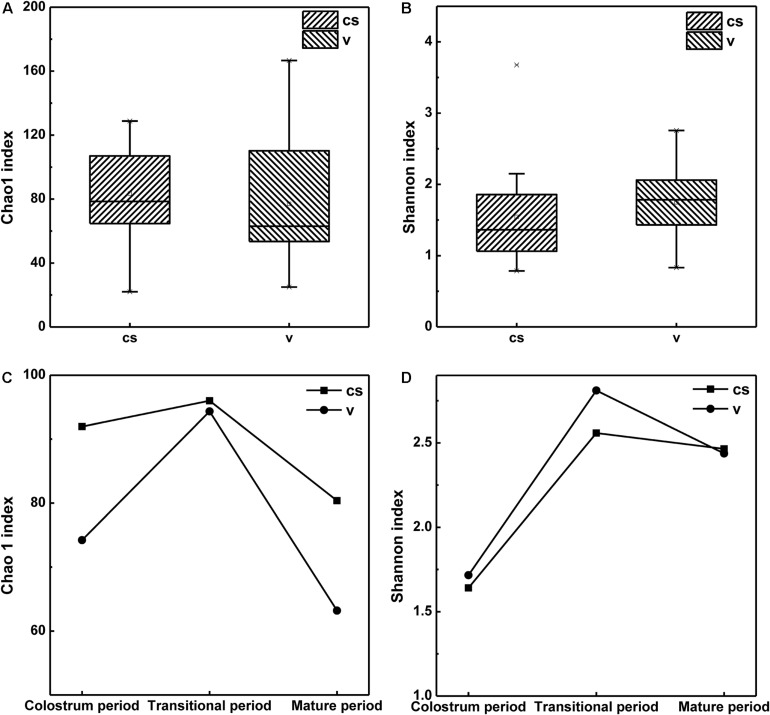
Alpha diversity of the stool microbiota of infant born via different delivery modes. **(A)** The Chao1 index of infant stool. **(B)** The Shannon index of infant stool. **(C)** Changes in Chao1 index in the stool samples of infants in different stages. **(D)** Changes in Shannon index in the stool samples of infants in different stages.

Within-sample (alpha) diversity of the infant stool samples was assessed using two indices, namely, the Shannon and Chao1 indices. As shown in Figure 1A, for all samples, the microbiota of C-section infants exhibited higher Chao1 index than those of vaginal delivery infants; however, the difference was not significant. This indicated that the infants in the vaginal delivery group had a higher number of bacterial species than the infants in the C-section group. Meanwhile, the microbiota in the stool samples of C-section infants showed a non-significantly lower Shannon index than those in stool samples of vaginal delivery infants (Supplementary Table 1). This result suggested that vaginal delivery had higher evenness in infant stool (Figure 1B).

All samples were grouped according to infants’ age: 1–7 days (colostrum period), 8–14 days (transitional period) and 15–30 days (mature period). The results also indicated that after birth, the bacterial species in the infant stool had increased from the 1st day to the 14th day after birth and then declined until the 30th day. Shannon index also showed a similar trend, indicating that the evenness of the intestinal microbiota was richest during the transitional period and then decreased during the maturation period. Both Chao1 and Shannon indices showed the highest values during the transitional period, whether in the stool samples of C-section infants or in those of vaginal delivery infants (Figures 1C,D). This meant that there were more species and bacterial homogeneity in the transitional period than in the other periods both in the C-section and vaginal delivery modes.

### Bacterial Components in the Infant’s Intestine Between the Two Delivery Modes

**FIGURE 2:**
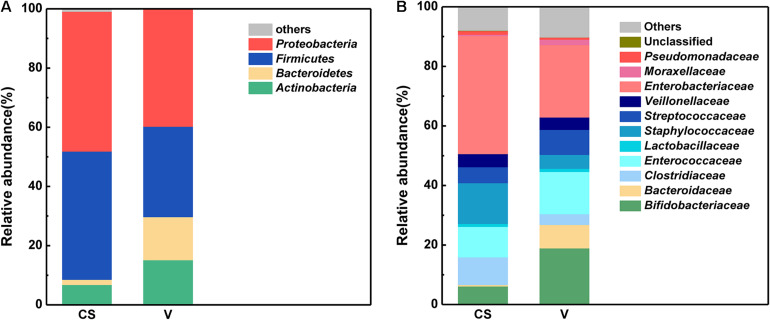
Characteristics of the infant stool microbiota between the two different delivery modes at the phylum and family levels. **(A)** Phylum level characteristics of infant stool microbiota between the two different delivery modes. **(B)** Family level characteristics of infant stool microbiota between the two different delivery modes.

The relative abundance of the different bacteria in the infants born via the two delivery modes is depicted in Figure 2. Proteobacteria and Firmicutes were the dominant phyla observed in these two delivery modes (Figure 2A). Further, the number of Proteobacteria and Firmicutes was higher in the C-section group than in the vaginal delivery group. In contrast, Bacteroidetes *(Bacteroidaceae)* and Actinobacteria *(Bifidobacteriaceae)* occupied only a small percentage compared with the above two phyla. At the family level (Figure 2B), *Enterobacteriaceae* exhibited the highest relative abundance in the C-section group, followed by *Staphylococcaceae and Lactobacillaceae*. In contrast, *Enterobacteriaceae*, *Bifidobacteriaceae*, and *Lactobacillaceae* were dominant in the vaginal delivery group, and other families only occupied a small percentage.

**FIGURE 3:**
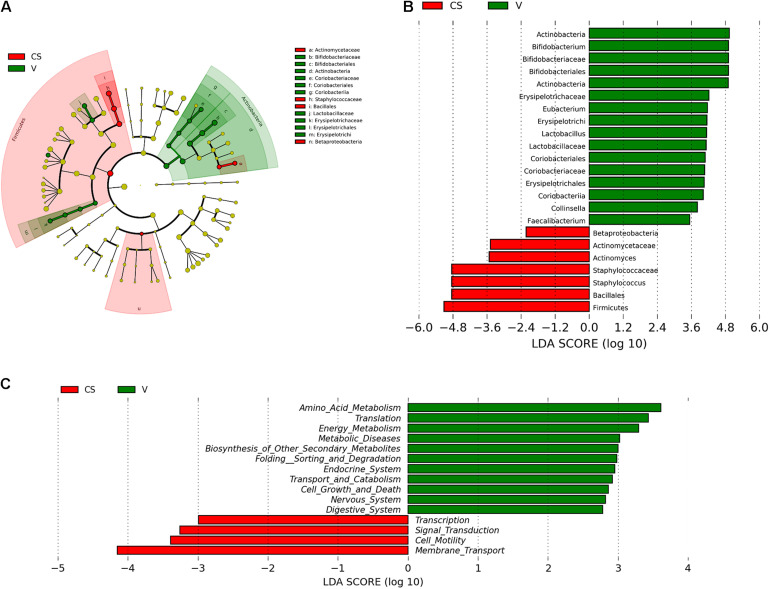
Comparison of the different microbiota in the stool samples of infants born via different delivery modes. **(A)** LEfSe analysis of the different microbiota in stool samples of vaginal delivery and C-section infants. Microbial communities in the samples of the vaginal delivery infants via their LDA value statistics are shown in green; C-section samples with a negative LDA score are shown in red. LDA value is absolute. **(B)** Cladogram derived from LEfSe analysis of the metagenomic sequences from the stool samples of C-section and vaginal delivery infants. Green areas indicate a significant effect on the microbial groups in the vaginal delivery group. Red areas indicate a significant effect on the microbial groups in the C-section group. LDA values were obtained via linear regression analysis. **(C)** Analysis of KEGG differential pathways via LEfSe.

LEfSe and cladistics analyses were performed to investigate the differences in the community composition between the two different delivery modes. At the phylum level, Actinobacteria and Firmicutes were dominant in both groups. At the family level, there were five different families, with enrichment of *Bifidobacteriaceae*, *Erysipelotrichaceae*, *Lactobacillaceae*, *Coribacteriaceae*, and *Coribacteriaceae* in the vaginal delivery group and that of *Actinomycetaceae* and *Staphylococcaceae* in the C-section group (Figures 3A,B). There were seven different genera between the two groups. *Bifidobacterium*, *Eubacterium*, *Lactobacillus*, *Collinsella*, and *Faecalibacterium* exhibited a relatively high abundance in the vaginal delivery group, whereas *Actinomyces* and *Staphylococcus* were more abundant in the C-section group (Figures 3A,B).

### Function Prediction of Infant’s Intestinal Microbiota Between the Two Delivery Modes

LEfSe analysis was performed to analyze the different functions between the two delivery modes. The abscissa is the log value of the value obtained via linear regression analysis (LDA), as depicted in Figure 3C. According to function prediction of the microbiota (Figure 3C), it could be concluded that the infant stool microbiota in the C-section group was related to membrane transport, cell motility, signal transduction and transcription. In contrast, the infant stool microbiota in the vaginal delivery group was associated with the digestive system.

### Strain Isolation

Semi-selective medium was used to isolate the target strains from 25 vaginal-delivered infant stool samples. A total of 95 strains were isolated from 25 vaginal-delivered infant stool samples. Among them, 40 putative *Lactobacillus* and *Bifidobacterium* strains were screened via microscopic examination and colony morphology analysis.

### Surface Hydrophobicity of the Isolates

**FIGURE 4:**
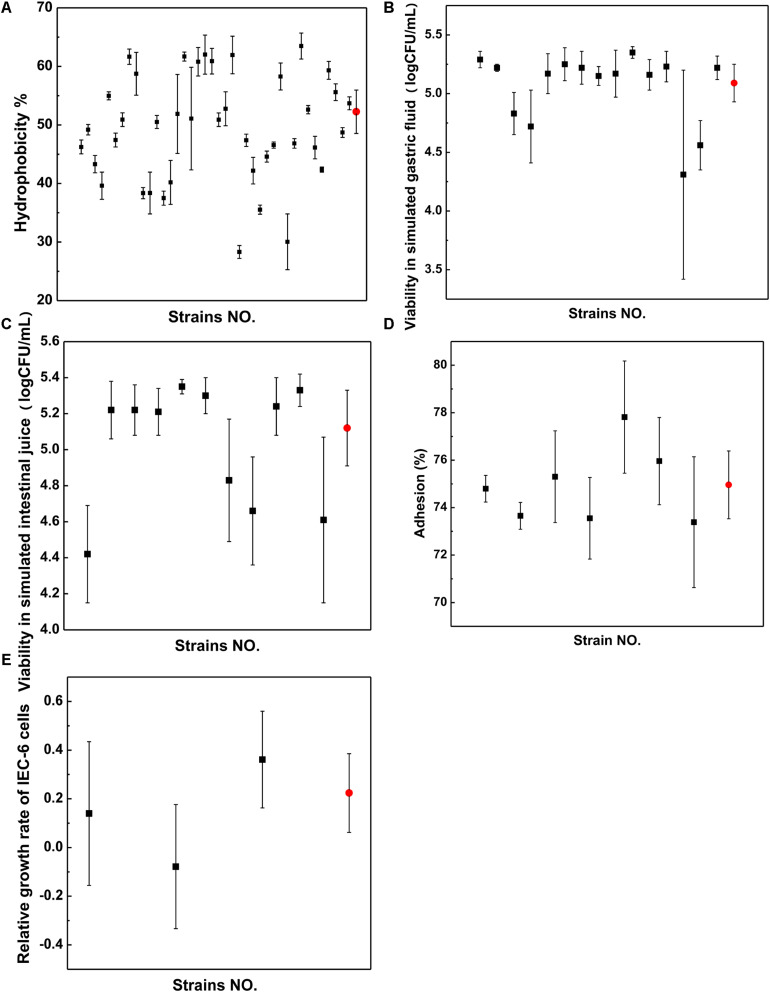
Properties of strains. **(A)** Surface hydrophobicity. **(B)** Viability in simulated gastric fluid. **(C)** Viability in simulated intestinal juice. **(D)** Adhesion ability. **(E)** Relative growth rate. Black dots represent strains from infant stool samples and red dots represent those from LGG.

The hydrophobicity method was used to screen the strains, as shown in Figure 4A. The surface hydrophobicity of the 40 putatively differential strains ranged from 28.31 to 63.47%, as shown in Figure 4A. Among them, 15 strains were screened because they had higher hydrophobicity percentages than the reference strain LGG (52.26%); in particular, the strain named FL-228.1 exhibited the highest hydrophobicity (63.47%). On average, *Lactobacillus* (51.62%) showed better hydrophobicity than *Bifidobacterium* (47.61%).

### Gastrointestinal Tract Viability of the Isolates

The survival rates of 11 strains showed higher resistance to acidic conditions than LGG and were screened following exposure to simulated gastric fluid from the 15 strains, as shown in Figure 4B. The survival rates of seven strains were screened following exposure to simulated intestinal juice from 11 strains, as shown in Figure 4C. The results indicated that compared with LGG, the seven strains showed higher resistance to the simulated intestinal juice condition. The isolate FL-228.1 showed the highest resistance. A total of seven strains were screened for surface hydrophobicity and gastrointestinal tolerance indicator.

### Adhesion Ability of the Different Strains to IEC-6 Cells

Through gastrointestinal fluid simulation and hydrophobicity experiments, seven bacterial strains were screened for further cell adhesion tests. Compared with LGG, three isolates from the seven strains, namely, FL-215, FL-216.9 and FL-228.1, showed higher adhesion abilities to IEC-6 cells. Further, among the three isolates, FL-228.1 showed the strongest adhesion ability to IEC-6 cells, as shown in Figure 4D. The specific results of the adhesion ability of the different strains are shown in Supplementary Table 2.

### Effect of the Isolates on the Development of Intestinal Epithelial Cells *in vitro*

Through cell adhesion tests, three bacterial strains were screened for promoting the development of intestinal epithelial cells. The *in vitro* study revealed that FL-215 and FL-228.1 promoted the development of intestinal epithelial cells and that FL-216.9 could not function in promoting the development of intestinal epithelial cells. Further, FL-228.1 showed improvements compared with the LGG group in terms of the relative growth rate of IEC-6 cells (Figure 4E).

### Effect of *B. bifidum* FL-228.1 on the Intestinal Morphological Characteristics of Rat Pups

**FIGURE 5:**
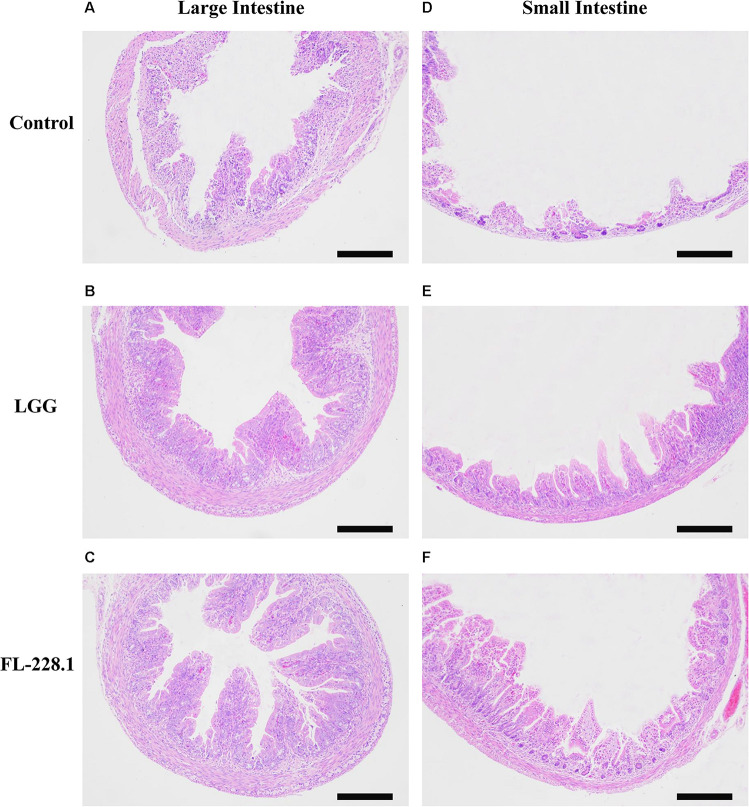
The intestinal morphological characteristics of the different groups based on hematoxylin–eosin (H&E) staining (all scale bar, 100 μm). **(A)** Morphology of the large intestine of the pups in the blank control group. **(B)** Morphology of the large intestine of the pups fed with the LGG strain. **(C)** Morphology of the large intestine of the pups fed with the FL-228.1 strain. **(D)** Morphology of the small intestine of the pups in the blank control group. **(E)** Morphology of the small intestine of the pups fed with the LGG strain. **(F)** Morphology of the small intestine of the pups fed with the FL-228.1 strain.

The morphological characteristics of the different parts of the rat pup’s intestine after intervention with the FL-228.1 strain for 10 days are depicted in Figure 5. Compared with the blank control group and LGG, the pups fed with FL-228.1 exhibited longer large intestine villi (Figures 5A–C). Furthermore, FL-228.1 exhibited longer small intestine villi in the offspring compared with blank control group and LGG (Figures 5D–F). Therefore, FL-228.1 promoted the development of small and large intestinal villi in the offspring.

### Effect of *B. bifidum* FL-228.1 on Intestinal Cell Development in the Rat Pups

**FIGURE 6:**
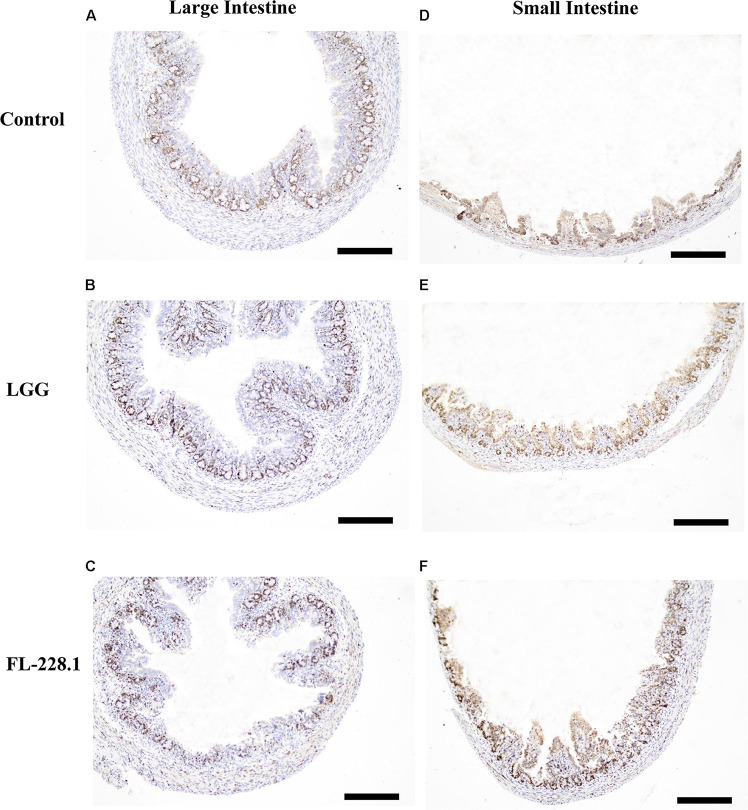
Intestinal cell development in the different groups based on immunostaining for Ki67 (all scale bar, 100 μm). **(A)** Morphology of the large intestine of the pups in the blank control group. **(B)** Morphology of the large intestine of the pups fed with the LGG strain. **(C)** Morphology of the large intestine of the pups fed with the FL-228.1 strain. **(D)** Morphology of the small intestine of the pups in the blank control group. **(E)** Morphology of the small intestine of the pups fed with the LGG strain. **(F)** Morphology of the small intestine of the pups fed with the FL-228.1 strain.

**FIGURE 7:**
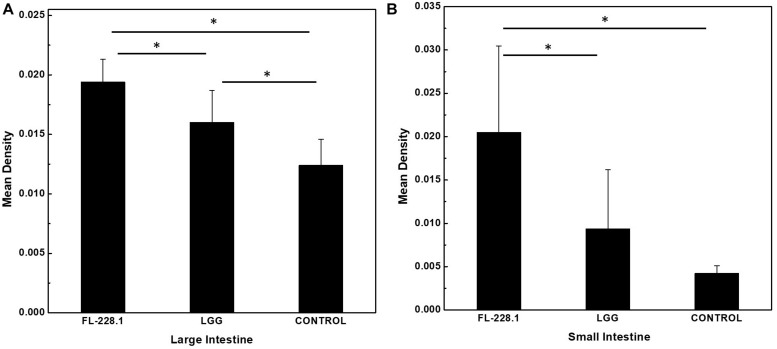
The mean density of the small and large intestines of the different groups. **(A)** The mean density of the large intestine of the different groups. **(B)** The mean density of the small intestine of the different groups. *P < 0.05.

Following intervention with the FL-228.1 strain for 10 days, intestinal cell development in the rat pups is depicted in Figures 6, 7. Compared with the blank and LGG groups, the mean densities of the large intestine and small intestine of the rats fed with FL-228.1 were the highest. Furthermore, for the large intestine, the mean density of the FL-228.1-fed pups was significantly higher than that of LGG-fed or blank groups (*P* < 0.05). The mean density of the LGG-fed pups was significantly higher than that of the control group (*P* < 0.05) (Figure 7A). Moreover, in the small intestine, the mean density of the FL-228.1-fed pups was significantly higher than that of LGG-fed and blank groups (*P* < 0.05) (Figure 7B). Therefore, FL-228.1 can promote intestinal cell development in the small and large intestines of the offspring.

## Discussion

### Effect of Different Delivery Modes on the Composition of Infant Intestinal Microbiota

The 16S rRNA gene Illumina HiSeq platform was used to analyze stool samples from healthy infants. Although our study was limited by sequencing depth and length, to our knowledge, our study was the first to investigate the microbial diversity of infant stool samples from two delivery methods from subjects living in northeast China.

We observed some associations among stool microbiota, community composition and modes of delivery (Figure 2). At the phylum level, the most abundant bacterial phyla were Firmicutes and Proteobacteria in these two delivery modes. This result is consistent with that of a recent investigation in another cohort (Wampach et al., 2018). Moreover, the most apparent differences at the family level were the relative abundances of *Bifidobacteriaceae*, which were dominant in the vaginal delivery group. This result was generally consistent with that of previous studies, which found a greater relative abundance of *Bifidobacteriaceae* in the stool samples of vaginal delivery infants (Shao et al., 2019). In contrast, the relative abundance of *Clostridiaceae* and *Staphylococcaceae* was higher in the stool samples of C-section infants presented by mother’s skin and the hospital environment (Madan et al., 2016a; Tamburini et al., 2016; Shi et al., 2018). Moreover, samples from vaginal delivery infants were enriched with *Bacteroidaceae*; this result is consistent with that of a recent investigation in another cohort (Wampach et al., 2018).

There were apparent differences in infant intestinal microbiota in the two different delivery modes. According to the results of biodiversity analysis (Figures 3A,B), we found that *Bifidobacterium* and *Lactobacillus* were the different intestinal microbiota in the vaginal delivery and C-section groups. Meanwhile, according to the results of LEfSe analysis and function prediction, it was speculated that different bacterial groups lead to different functions. The vaginal delivery group was associated with the digestive system and cell growth and death. Function prediction could provide guidance for subsequent bacterial isolation and functional verification. Therefore, we analyzed the potential efficacies of these two genera, which are anaerobic and gram-positive. Meanwhile, many studies have confirmed that *Bifidobacterium* and *Lactobacillus* have certain effects on intestinal development (Mi et al., 2017; O’Connell Motherway et al., 2019; Zhou et al., 2020). Therefore, we analyzed only anaerobic and gram-positive bacterial strains and used *Bifidobacterium* and *Lactobacillus* as our target strains.

### Bacterial Strains Promote Intestinal Development

*Bifidobacterium* and *Lactobacillus* had important effects on intestinal development (Blanton et al., 2016; Mi et al., 2017; O’Connell Motherway et al., 2019; Zhou et al., 2020). The tight adhesion (Tad) pili of *Bifidobacterium* is an important colonizing factor that promotes the development of intestinal epithelial cells (O’Connell Motherway et al., 2019). It was confirmed that the development of intestinal epithelial cells significantly increased 5 days after *Bifidobacterium* ingestion in mice. Furthermore, the Tad pili of *Bifidobacterium* promoted the intestinal mucosal growth of neonates by producing specific extracellular protein scaffolds, thereby promoting intestinal maturation of early neonates (O’Connell Motherway et al., 2019). This result is consistent with our results as FL-228.1 exhibited greater ability to develop in the intestine than the LGG and blank control group. Another study demonstrated that *Bif. longum* can enhance intestinal development and mucosal repair, promote lysozyme production and ameliorate dysbiosis of the microbiota in WAS rats by upregulating the stem niche factors WNT3A and TGF-β, which are secreted by Paneth cells (Zhou et al., 2020). Mi et al. (2017) studied the effects of *B. infantis* in attenuating the severity of chemotherapy-induced intestinal mucositis. They demonstrated that the *B*. *infantis* group showed higher intestinal villus height and deeper crypt depth than the chemotherapy group. It has been shown that LGG can promote intestinal epithelial homeostasis through specific signaling pathways. Researchers have also found that two proteins secreted from LGG, i.e., p75 (75 Dalton) and P40 (40 Dalton), promote the growth of the human and mouse colonic epithelial cells. At the same time, it promotes the growth of mouse colon cells *in vitro*. The current study screened one *B. bifidum* FL-228.1 strain that exhibited a higher ability to promote intestinal development than LGG. Overall, the results of this study are consistent with those of previous studies. However, previous studies have only evaluated the function of the intestinal microbiota and the mechanism involved in promoting the development of intestinal epithelial cells. This study was more specific and might be beneficial to the study of probiotic effects in the future. Further research should also focus on specific probiotic functions.

## Data Availability Statement

The datasets presented in this study can be found in online repositories. The names of the repository/repositories and accession number(s) can be found in the article/Supplementary Material.

## Ethics Statement

All procedures performed in studies involving human participants were in accordance with the ethical standards of the institutional (HRYLL201607). Written informed consent to participate in this study was provided by the participants’ legal guardian/next of kin. Written informed consent was obtained from the individual(s), and minor(s)’ legal guardian/next of kin, for the publication of any potentially identifiable images or data included in this article.

## Author Contributions

LZL: writing-original draft preparation, formal analysis, and investigation. XZ, MZ, LL, SJ, JP, and YL: samples collections. PG: reviewing and editing. HN, JZ, and SC: investigation and data curation. XH: project administration. LZ and DC: funding acquisition, supervision, conceptualization, writing–review, and editing. All authors contributed to the article and approved the submitted version.

## Conflict of Interest

SJ, JP, and YL were employed by Heilongjiang Feihe Dairy Co., Ltd. The remaining authors declare that the research was conducted in the absence of any commercial or financial relationships that could be construed as a potential conflict of interest.

## Publisher’s Note

All claims expressed in this article are solely those of the authors and do not necessarily represent those of their affiliated organizations, or those of the publisher, the editors and the reviewers. Any product that may be evaluated in this article, or claim that may be made by its manufacturer, is not guaranteed or endorsed by the publisher.

## Footnotes

1
http://www.ncbi.nlm.nih.gov
