# Supplementary material for: Delivery Mode Affects Intestinal Microbial Composition and the Development of Intestinal Epithelial Cells
Source: Front Microbiol. 2021 Aug 13;12:626144. doi: 10.3389/fmicb.2021.626144 (PMC8414977; doi:10.3389/fmicb.2021.626144)
Supplement: Supplementary file 1 [file Data_Sheet_1.docx]

**Supplementary material**

Table S1 Chao 1 and Shannon index in infant stool

| Samples  No. | C-section | | Samples  No. | Vaginal delivery | |
| --- | --- | --- | --- | --- | --- |
|  | Chao 1  index | Shannon index |  | Chao 1 index | Shannon index |
| 1 | 46.00 | 1.35 | 1 | 134.00 | 2.76 |
| 2 | 75.20 | 1.75 | 2 | 53.50 | 1.85 |
| 3 | 119.00 | 1.05 | 3 | 133.33 | 2.06 |
| 4 | 87.25 | 1.42 | 4 | 101.25 | 1.94 |
| 5 | 64.00 | 1.29 | 5 | 55.50 | 2.04 |
| 6 | 76.75 | 1.06 | 6 | 58.11 | 1.36 |
| 7 | 95.63 | 1.25 | 7 | 63.00 | 0.83 |
| 8 | 57.00 | 1.38 | 8 | 110.75 | 1.55 |
| 9 | 77.75 | 1.87 | 9 | 110.20 | 2.20 |
| 10 | 102.20 | 2.15 | 10 | 124.80 | 1.78 |
| 11 | 89.11 | 1.80 | 11 | 54.63 | 2.11 |
| 12 | 48.11 | 0.99 | 12 | 115.20 | 2.34 |
| 13 | 116.23 | 1.86 | 13 | 79.20 | 1.73 |
| 14 | 110.25 | 1.91 | 14 | 86.50 | 1.54 |
| 15 | 107.00 | 1.17 | 15 | 166.67 | 1.09 |
| 16 | 116.11 | 1.07 | 16 | 56.33 | 2.27 |
| 17 | 74.67 | 1.23 | 17 | 60.25 | 1.96 |
| 18 | 100.67 | 1.90 | 18 | 38.00 | 2.20 |
| 19 | 68.00 | 0.90 | 19 | 45.50 | 1.26 |
| 20 | 69.67 | 1.72 | 20 | 69.00 | 1.46 |
| 21 | 64.67 | 1.40 | 21 | 28.00 | 1.43 |
| 22 | 54.60 | 0.82 | 22 | 29.00 | 1.36 |
| 23 | 128.75 | 3.68 | 23 | 45.33 | 1.21 |
| 24 | 119.50 | 3.38 | 24 | 25.00 | 1.44 |
| 25 | 22.00 | 0.95 | 25 | 80.60 | 1.92 |
| 26 | 79.30 | 0.79 |  |  |  |
| Mean±SD | 83.44±27.00 | 1.54±0.70 | Mean±SD | 76.95±38.14 | 1.75±0.45 |

Table S2 Adhesion abilities

| Strains No. | Adhesion abilities (log CFU/mL) | | |  |
| --- | --- | --- | --- | --- |
|  | 1 | 2 | 3 | Mean±SD |
| FL-76 | 6.03 | 5.94 | 5.98 | 5.98±0.05 |
| FL-135 | 5.87 | 5.86 | 5.94 | 5.89±0.04 |
| FL-215 | 5.85 | 6.15 | 6.07 | 6.02±0.16 |
| FL-215.1 | 5.79 | 5.83 | 6.04 | 5.88±0.13 |
| FL-216.9 | 6.23 | 6.03 | 6.41 | 6.23±0.19 |
| FL-228.1 | 6.18 | 5.91 | 6.14 | 6.08±0.15 |
| FL-438 | 6.05 | 5.94 | 5.62 | 5.87±0.22 |
| LGG | 6.13 | 5.91 | 5.95 | 6.00±0.12 |
